# Concordance in Medical Urgency Classification of Discharge Diagnoses and Reasons for Visit

**DOI:** 10.1001/jamanetworkopen.2023.50522

**Published:** 2024-01-10

**Authors:** Theodoros V. Giannouchos, Benjamin Ukert, Brad Wright

**Affiliations:** 1 Department of Health Policy and Organization, School of Public Health, The University of Alabama at Birmingham; 2 Department of Health Policy and Management, School of Public Health, Texas A&M University, College Station; 3 Department of Health Services Policy and Management, Arnold School of Public Health, University of South Carolina, Columbia

## Abstract

**Importance:**

Current policies to divert emergency department (ED) visits for less medically urgent conditions to more cost-effective settings rely on retrospective adjudication of discharge diagnoses. However, patients present to the ED with concerns, making it challenging for clinicians.

**Objective:**

To characterize ED visits based on the medical urgency of the presenting reasons for visit and to explore the concordance between discharge diagnoses and reasons for visit.

**Design, Setting, and Participants:**

In this retrospective, cross-sectional study, a nationwide sample of ED visits by adults (aged ≥18 years) in the US from the 2018 and 2019 calendar years’ ED data of the National Hospital Ambulatory Medical Care Survey was used. An algorithm to probabilistically assign ED visits into medical urgency categories based on the presenting reason for visit was developed. A 3-step, look-back method was applied using an updated version of the New York University ED algorithm, and a map of all possible discharge diagnoses to the same reasons for visit was developed. Analyses were conducted in July and August 2023.

**Main Outcomes and Measures:**

The main outcome was probabilistic medical urgency classification of reasons for visits and discharge diagnoses and their concordance.

**Results:**

We analyzed 27 068 ED visits (mean age, 48.2% years [95% CI, 47.5%-48.9% years]) representing 190.7 million visits nationwide. Women (mean, 57.0% [95% CI, 55.9%-58.1%]) and patients with public health insurance coverage, including Medicare (mean, 24.9% [95% CI, 21.9%-28.0%]) and Medicaid (mean, 25.1% [95% CI, 21.0%-29.2%]), accounted for the largest share of ED visits, and a mean of 13.2% (95% CI, 11.4%-15.0%) of all visits resulted in a hospital admission. Overall, about 38.5% and 53.9% of all ED visits were classified with 100% and 75% probabilities, respectively, as injury related, emergency care needed, emergent but primary care treatable, nonemergent, or mental health or substance use disorders related based on discharge diagnosis compared with 0.4% and 12.4%, respectively, of all encounters based on patients’ reason for visit. Among discharge diagnoses assigned with high certainty to only 1 urgency category using the New York University ED algorithm, between 38.0% (95% CI, 36.3%-39.6%) and 57.4% (95% CI, 56.0%-58.8%) aligned with the probabilistic categorical assignments of their corresponding reasons for visit.

**Conclusions and Relevance:**

In this cross-sectional study of 190.7 million ED visits among adults aged 18 years or older, a smaller percentage of reasons for visit could be prospectively categorized with high accuracy to a specific medical urgency category compared with all visits based on discharge diagnoses, and a limited concordance between reasons for visit and discharge diagnoses was found. Alternative methods are needed to identify the medical necessity of ED encounters more accurately.

## Key Points

**Question:**

What is the concordance in medical urgency classifications between discharge diagnoses and the corresponding presenting reasons for visit in emergency department (ED) settings?

**Findings:**

In this cross-sectional study of 190.7 million ED visits among adults aged 18 years or older, after mapping all possible discharge diagnoses to the same reasons for visit, 12.4% of reasons for visit could be prospectively categorized with high accuracy to a specific medical urgency category compared with 53.9% of all visits based on discharge diagnoses, while reasons for visits also had limited concordance with discharge diagnoses.

**Meaning:**

The high uncertainty of the required care at the time of arrival at the ED suggests that strategies to minimize less medically urgent ED visits based on postdischarge diagnoses are questionable, emphasizing the need for alternative methods to identify nonemergent encounters.

## Teaser

This cross-sectional study assesses the concordance in medical urgency classifications between discharge diagnoses and the corresponding presenting reasons for visit in emergency department (ED) settings based on data in the US in 2018 and 2019.

## Introduction

Each year, around 140 million emergency department (ED) visits occur in the US, at a rate of 4 visits per 10 people and totaling almost $80 billion in spending.^1,2,3^ Current estimates suggest that nearly 40% of all ED visits are medically nonurgent.^4,5,6,7,8,9^ Emergency departments are ill-equipped to provide definitive care and long-term monitoring and management for patients seeking ED care with such nonurgent conditions; EDs are also high-cost and resource-intensive environments. Thus, diverting ED visits for less medically urgent conditions to more cost-effective settings, like physicians’ offices and urgent care centers, may present a substantial opportunity to improve health outcomes and contain spending.^9,10^

Various interventions to reduce medically nonurgent ED visits have been suggested, pilot tested, and evaluated nationwide, ranging from expanding health insurance coverage to financial incentives, patient education, case management, care coordination, and enhanced primary care access.^11,12,13,14,15,16,17,18^ State legislatures as well as private and public health insurers have also attempted to reduce ED visits by deeming some encounters as nonemergent or unnecessary and limiting reimbursement, implementing copayments, and denying coverage.^14,19,20,21,22,23,24,25^

However, initiatives aimed at reducing ED use for medically nonurgent conditions have been criticized for the way in which they identify and classify ED visits as nonurgent and potentially avoidable.^23,24,25^ Currently, the medical necessity of ED visits, which guides decision-making and shapes policies, relies on retrospective review and adjudication of ED visits using medical claims and algorithms based on discharge diagnoses.^3,6,7,9,23,24,25,26,27,28^ Yet patients present to the ED with concerns and symptoms, not a discharge diagnosis, and are ill-equipped to accurately assess the severity of their medical problem in advance.^23,24,25,28^ Given the limited information available when patients first arrive at the ED, the clinical decision-making guidelines, and medical complexity, it can also be challenging for clinicians to accurately identify a discharge diagnosis based on the patient’s reasons for seeking ED care at the time of arrival.^23,24,25^

Previous work has found that almost 90% of ED visits classified retrospectively as medically nonurgent had the same reason for visit as emergent visits, and almost one-third of these visits were triaged as urgent upon arrival.^24^ These findings highlight risk-related and clinically related uncertainties between both patients and clinicians at the initial stage of ED presentation, since it is often not obvious whether the visit will result in a nonemergent or emergent case. Hence, using discharge diagnoses to guide policy implementation and evaluation may be problematic. Despite the important contributions of existing studies on medical urgency of ED use, almost all studies rely on retrospective algorithms for ED visit classification based on discharge diagnoses. Moreover, existing studies analyzing ED visits by presenting reasons and concerns, to our knowledge, examined only nonemergent encounters, which account for a small share of all ED visits, and relied on data that were nearly 1 decade old.^24,25^

To address this gap in the literature, we used a nationwide sample of ED visits from 2018 and 2019 in the US to characterize all ED visits based on the medical urgency of the presenting reasons for visit. To do so, we developed an algorithm to probabilistically assign ED visits into categories indicating medical urgency based on the presenting reason for visit. We hypothesized that the urgency level of an ED visit from the discharge diagnosis would differ and might thus be a poor predictor when compared with the urgency level identified from the reason for visit. As policymakers and stakeholders contemplate initiatives to reduce ED visits and lower spending, our findings can inform future policy by documenting the extent of clinical uncertainty at patients’ initial presentation to the ED and by underscoring the limitations inherent in the current method of retrospectively adjudicating ED visits.

## Methods

### Study Design, Population, and Data Source

We conducted a retrospective, cross-sectional study of ED visits by all adults (aged ≥18 years) in the US using the 2018 and 2019 calendar years’ ED microdata files of the National Hospital Ambulatory Medical Care Survey (NHAMCS).^29^ We followed the Strengthening the Reporting of Observational Studies in Epidemiology (STROBE) reporting guideline for cross-sectional studies.^30^ The NHAMCS is a 3-stage, probabilistic survey based on a national sample of visits to EDs in noninstitutional general and short-stay hospitals across all states and the District of Columbia, excluding federal military and Veterans Administration hospitals. Participating EDs are randomly assigned to a 4-week reporting period, and sociodemographic, clinical, and visit-level information is collected from patients’ medical records by trained staff on a systematic, random sample of ED visits using structured forms. The study was determined not to be human participants research by the University of South Carolina institutional review board. The databases are publicly available and use deidentified patient data; informed consent was not required.

Race and ethnicity categories included Hispanic, non-Hispanic Black, non-Hispanic White, and non-Hispanic Other (including American Indian or Alaska Native, Asian, Native Hawaiian or Other Pacific Islander, and those reporting more than 1 race or ethnicity). These data were included for descriptive purposes only, as about 1 in every 7 ED visits in the data included a regression-adjusted imputed category due to missing information from patient records; we did not make any adjustment for race and ethnicity using multivariable models or conduct any stratified analyses by race and ethnicity.

### Algorithmic Development and Probabilistic Classification of Reasons for Visit by Medical Urgency

Our main outcome of interest was the medical urgency classification of the presenting reason for each ED visit. The NHAMCS included patients’ reasons for visit, documented as verbatim text by triage nurses, which were classified and categorized by trained abstractors using the reason-for-visit classification system in the NHAMCS. However, since it was not possible to classify patients’ reasons for ED visits according to their medical urgency using the reason-for-visit classification system, we devised a method for assigning medical urgency probabilities to patients’ reasons for visiting the ED.

To do so, we used a 3-step, look-back method to probabilistically classify reasons for visits by medical urgency, guided by previous work (Figure).^24^ First, we used the updated version of the New York University (NYU) ED algorithm, a tool commonly used by policymakers and researchers to assign probabilities to discharge diagnoses for ED visits based on the *International Statistical Classification of Diseases and Related Health Problems, Tenth Revision* codes.^5^ This algorithm initially identifies discharge diagnoses associated with injuries, mental health, alcohol, and substance use disorders and assigns a 100% probability to those diagnoses. For the remaining diagnoses, the algorithm assigns probabilities and classifies them into 4 nonmutually exclusive categories as nonemergent, emergent but primary care treatable, emergent with needed ED care but preventable or avoidable, and emergent with needed ED care and not preventable or avoidable. The original algorithm was developed by a panel of ED physicians and researchers after reviewing a sample of around 6000 ED records and applying probabilistic weights to discharge diagnoses across the 8 categories.^31^ The updated algorithm used in this study was designed to broaden the external validity of the original algorithm by reducing the number of unclassifiable diagnoses, rather than improving its accuracy.^31^ For example, in the NYU ED algorithm, acute myocardial infarction (heart attack) is assigned a 100% probability of being emergent with needed ED care and not preventable or avoidable, while chronic serous otitis media is assigned a 100% probability of being nonemergent. However, in some cases, probabilities are assigned to multiple categories (eg, thoracic spine pain: 66.6% not preventable or avoidable; 33.4% primary care treatable). We then combined the NYU ED algorithm categories into 5 groups indicating medical urgency, namely as injuries, ED care needed (ED care need and not preventable or avoidable, ED care need but preventable or avoidable), emergent but primary care treatable, nonemergent, and mental health or substance use disorder related.

**Figure.**
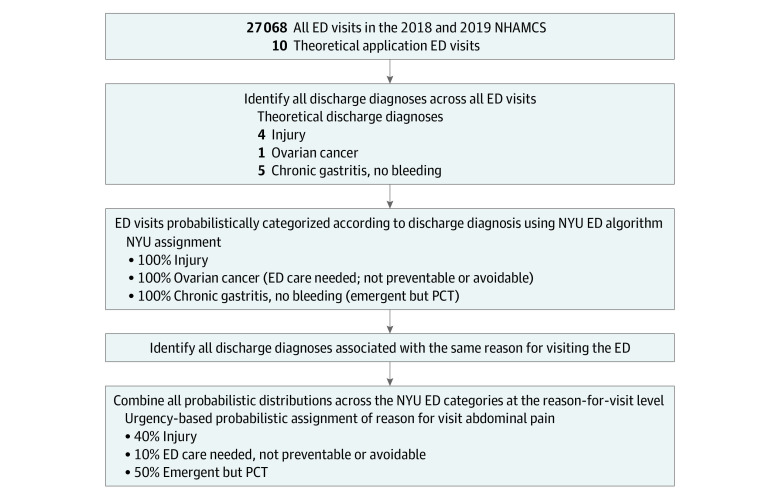
Algorithm for Probabilistic Classification of Emergency Department (ED) Visits by Medical Urgency Based on Reasons for Visit NHAMCS indicates National Hospital Ambulatory Medical Care Survey; NYU, New York University; PCT, primary care treatable.

In the second step, we identified and mapped all discharge diagnoses associated with the same reason for visit to create probabilistic distributions for each reason for visit. Different discharge diagnoses can be assigned across 1 or multiple categories with different probabilities by the NYU ED algorithm but might have the same reason for visit. In the third step, we assigned a weighted mean probabilistic classification from all documented discharge diagnoses to each reason for the ED visit. For example, patients presenting to the ED with abdominal pain may be discharged with a diagnosis for injury, a urinary tract infection, gastritis, or liver cancer. Hence, any ED visit with abdominal pain could be classified as 40% injury, 10% emergent and not preventable, and 50% emergent but preventable or avoidable (Figure). This approach resulted in the same 5 numeric categories as the NYU ED assignment, but in many instances, these were not mutually exclusive. Encounters with missing reasons for visit or discharge diagnoses and unclassifiable encounters by the NYU algorithm were excluded. This resulted in 27 068 ED visits representing 190.7 million visits after survey weights were applied.

### Statistical Analysis

Analyses were conducted in July and August 2023. We initially described all ED visits’ sociodemographic, clinical, and encounter-level characteristics using mean frequencies and proportions and 95% CIs after applying survey weights available in the data to produce national estimates.^29^ We then identified ED visits with discharge diagnoses that were assigned into a specific medical urgency category with high certainty (ie, 100% nonemergent or 100% injury only) and compared those with the corresponding reasons-for-visit probabilistic assignment into the 5 outcome groups based on our algorithm. We anticipated variation in the probabilistic assignment across categories, given the patient-level and clinician-level uncertainties at the time of arrival to the ED compared with the discharge diagnoses, although the overall distributions of probabilities across categories were expected to be similar between discharge diagnoses and reasons for visit. Similarly, we examined the medical urgency categorization of the most common diagnoses based on the NYU ED algorithm and of the corresponding most common reasons for visit based on our 3-step, look-back method to identify potential differences in urgency classification. We validated potential differences of the probabilistic categorical assignment between the algorithms by examining the distributions of ED visits for any conditions that resulted in a hospital admission and visits that resulted in discharges from the ED with cerebral infarction (stroke) or acute myocardial infarction (heart attack) diagnoses. We expected that visits for stroke or heart attack and those resulting in admission would be assigned higher probabilities across more urgent categories (ie, emergency care needed) when using the post hoc discharge diagnosis. Data were managed using SAS, version 9.4 (SAS Institute Inc), and statistical analyses were conducted in Stata, version 17.0 (StataCorp LLC).

## Results

Our nationwide study included 190.7 million ED visits among adults aged 18 years or older occurring in 2018 and 2019 in the US. These visits were represented by 27 068 survey-weighted ED visits (mean age, 48.2% years [95% CI, 47.5%-48.9% years]) that were analyzed and included a mean of 43.0% (95% CI, 41.9%-44.1%) who were men and a mean of 57.0% (95% CI, 55.9%-58.1%) who were women. Public health insurance coverage, including Medicare (mean, 24.9% [95% CI, 21.9%-28.0%]) and Medicaid (mean, 25.1% [95% CI, 21.0%-29.2%]), accounted for the largest share of ED visits, and a mean of 13.2% [95% CI, 11.4%-15.0%]) of all visits resulted in a hospital admission. Descriptive characteristics of ED visits are presented in Table 1.

**Table 1.** Characteristics of All ED Visits Based on National Hospital Ambulatory Medical Care Survey 2018 and 2019 Data

Characteristic	All ED visits, mean, % (95% CI) (N = 190 680 594^a^)
**Sociodemographic characteristics**	
Age group, y	
18-24	12.6 (11.9-13.4)
25-34	18.4 (17.6-19.3)
35-44	16.1 (15.4-16.8)
45-54	15.0 (14.4-15.6)
55-64	14.7 (14.1-15.2)
≥65	23.2 (21.9-24.5)
Sex	
Female	57.0 (55.9-58.1)
Male	43.0 (41.9-44.1)
Race and ethnicity	
Hispanic	13.3 (11.4-15.6)
Non-Hispanic Black	24.1 (21.1-27.5)
Non-Hispanic White	59.1 (55.3-62.7)
Non-Hispanic other^b^	3.4 (2.7-4.3)
Health insurance	
Private	23.5 (20.3-26.7)
Medicare	24.9 (21.9-28.0)
Medicaid	25.1 (21.0-29.2)
Uninsured	9.8 (8.2-11.4)
Other	3.0 (2.4-3.6)
Unknown	13.7 (8.6-18.8)
Homeless	1.1 (0.9-1.4)
**Clinical characteristics**	
No. of chronic conditions	
0	38.6 (37.2-40.1)
1 or 2	38.0 (37.0-38.9)
≥3	23.4 (22.2-24.7)
Chronic conditions	
Alcohol or substance use disorder or dependence	10.8 (9.3-12.5)
Asthma	10.1 (9.4-10.7)
Cancer	4.9 (4.3-5.4)
Chronic obstructive pulmonary disease	7.8 (7.2-8.5)
Coronary artery disease	8.7 (8.1-9.3)
Depression	13.4 (12.4-14.5)
Diabetes	16.0 (15.3-16.8)
Hyperlipidemia	13.0 (11.9-14.1)
Hypertension	34.3 (33.1-35.4)
Obesity	7.1 (5.9-8.4)
**Visit-level characteristics**	
Admitted to hospital	13.2 (11.4-15.0)
Seen in this ED within the last 72 h	4.9 (4.1-5.8)
Weekend visit	25.7 (25.0-26.3)
After-hours visit	29.2 (27.8-30.7)
Arrival by ambulance	19.0 (17.8-20.3)
Left against medical advice, before being seen, or before treatment completion	2.2 (1.9-2.5)
MSA status	
MSA	83.7 (73.6-90.4)
Non-MSA	16.3 (9.6-26.4)
Geographic region	
Northeast	16.0 (12.8-19.9)
Midwest	19.5 (15.3-24.5)
South	43.0 (37.7-48.4)
West	21.5 (17.6-26.0)
Reason-for-visit algorithm	
Injury	19.4 (18.8-20.0)
ED care needed	27.9 (27.5-28.3)
Emergent but primary care treatable	24.7 (24.3-25.2)
Nonemergent	21.6 (21.1-22.1)
Mental health or substance use disorder	6.4 (6.0-6.7)
NYU ED algorithm	
Injury	18.7 (17.9-19.5)
ED care needed	28.8 (28.1-29.5)
Emergent but primary care treatable	24.5 (24.0-25.0)
Nonemergent	21.8 (21.0-22.5)
Mental health or substance use disorder	6.0 (5.5-6.5)
Immediacy in which the patient should be seen	
Unknown or no triage	27.6 (22.3-33.5)
Triage conducted	72.4 (66.5-77.7)
Immediate, emergent, or urgent	72.0 (68.3-75.9)
Semiurgent	24.9 (23.4-26.6)
Nonurgent	3.1 (2.3-4.0)

Abbreviations: ED, emergency department; MSA, Metropolitan Statistical Area; NYU, New York University.

^a^ N = 27 068 total weighted ED visits by adults (aged ≥18 years) in the US.

^b^ Non-Hispanic other category included American Indian or Alaska Native, Asian, Native Hawaiian or Other Pacific Islander, and those reporting more than 1 race.

The most common discharge diagnoses were unspecified or other chest pain (5.5%); unspecified abdominal pain (3.3%); pain in unspecified joint (2.3%); urinary tract infection (1.8%); headache (1.7%); and pain in limb, hand, foot, finger, or toes (1.5%) (eTable in Supplement 1). The most common reasons for visits were abdominal pain, cramps, or spasms (7.2%); chest pain or soreness (6.5%); shortness of breath (3.7%); nausea and/or vomiting (2.9%); headache or pain in the head (2.8%); and back pain, ache, soreness, or discomfort (2.8%) (eTable in Supplement 1).

### Classification by Medical Urgency Based on the Reason for Visit vs the NYU ED Algorithm

The classification of reasons for visit based on the developed algorithm suggested that the average reason for visit had more than a 70% probability of being a high-urgency encounter, with a 19.4% (95% CI, 18.8%-20.0%) probability of being an injury, a 27.9% (95% CI, 27.5%-28.3%) probability of being a visit with needed emergency care, and a 24.7% (95% CI, 24.3%-25.2%) probability of being an emergent but primary care–treatable visit (Table 1). The mean probability of being a nonemergent reason for visit was 21.6% (95% CI, 21.1%-22.1%), while another 6.4% (95% CI, 6.0%-6.7%) was associated with mental health or substance use disorders. Similar distributions were observed using the NYU ED algorithm. The mean probability of being an injury was 18.7% (95% CI, 17.9%-19.5%); a visit with needed emergency care, 28.8% (95% CI, 28.1%-29.5%); an emergent but primary care–treatable visit, 24.5% (95% CI, 24.-%-25.0%); a nonemergent reason, 21.8% (95% CI, 21.0%-22.5%); and associated with mental health or substance use disorders, 6.0% (95% CI, 5.5%-6.5%). Among ED visits that included triage information on how immediately the patient should be seen, 72.0% (95% CI, 68.3%-75.9%) were deemed as immediate, emergent, or urgent, and 24.9% (95% CI, 23.4%-26.6%) as semiurgent, while 3.1% (95% CI, 2.3%-4.0%) were initially triaged as being nonurgent.

Overall, of all ED visits categorized as injury related, emergency care needed, emergent but primary care treatable, nonemergent, or mental health or substance use disorders related, 38.5% (73.5 million) based on discharge diagnosis compared with 0.4% (790 713) based on their reason for visit were classified into a single category with 100% probability, and 53.9% (102.8 million) based on discharge diagnosis compared with 12.4% (23.5 million) of all encounters based on their reason for visit were classified with 75% probability (Table 2). Among discharge diagnoses assigned with high certainty to only 1 urgency category using the NYU ED algorithm, between a mean of 38.0% (95% CI, 36.3%-39.6%) and a mean of 57.4% (95% CI, 56.0%-58.8%) aligned with the probabilistic categorical assignments of their corresponding reasons for visit (Table 3). For example, among discharge diagnoses classified with 100% certainty as nonemergent, the mean probability of similarly being classified as nonemergent based on the reason at the time of the visit was 39.4% (95% CI, 38.1%-40.7%), while also having a mean probability of 12.5% (95% CI, 11.0%-14.0%) to be classified as injury-related, 20.5% (95% CI, 19.3%-21.7%) as ED care needed, and 25.7% (95% CI, 24.7%-26.8%) as an emergency but primary care–treatable based on the reasons for visit.

**Table 2.** Categorical Classification of ED Visits With High Accuracy Using the NYU Algorithm and the Developed RFV-Based Algorithm

Category	ED visits, No. (%) (N = 190 680 594)			
	NYU classification		RFV classification	
	With 100% probability	With 75% probability	With 100% probability	With 75% probability
Total	73 501 910 (38.5)	102 751 610 (53.9)	790 713 (0.4)	23 540 340 (12.4)
Injury	35 685 364 (18.7)	35 685 364 (18.7)	479 129 (0.3)	15 404 182 (8.1)
ED care needed	16 611 175 (8.7)	25 868 783 (13.6)	103 132 (0)	2 240 905 (1.2)
Emergent but PCT	3 690 768 (1.9)	14 938 609 (7.8)	59 967 (0)	91 834 (0)
Nonemergent	5 788 043 (3.0)	14 801 294 (7.8)	31 257 (0)	257 034 (0.1)
MH or SUD	11 457 560 (6.0)	11 457 560 (6.0)	117 228 (0)	5 546 385 (2.9)

Abbreviations: ED, emergency department; MH or SUD, mental health or substance use disorder; NYU, New York University; PCT, primary care treatable; RFV, reason for visit.

**Table 3.** Probabilistic Concordance Between the RFV-Based Algorithm and ED Visits Classified With Full Accuracy Into Categories Using the NYU Algorithm

NYU algorithm with 100% probabilistic assignment (No.)	RFV algorithm probabilistic concordance, mean % (95% CI)				
	Injury	ED care needed	Emergent but PCT	Nonemergent	MH or SUD
Injury (35 685 364)	57.4 (56.0-58.8)	11.7 (11.2-12.3)	11.6 (11.2-12.0)	16.1 (15.4-16.7)	3.2 (2.9-3.6)
ED care needed (16 611 175)	9.5 (8.6-10.5)	41.5 (40.4-42.6)	23.3 (22.5-24.1)	17.9 (17.3-18.5)	7.7 (6.8-8.7)
Emergent but PCT (3 690 768)	10.2 (8.2-12.2)	30.4 (28.4-32.4)	38.0 (36.3-39.6)	19.8 (18.0-21.6)	1.6 (1.2-2.1)
Nonemergent (5 788 043)	12.5 (11.0-14.0)	20.5 (19.3-21.7)	25.7 (24.7-26.8)	39.4 (38.1-40.7)	1.8 (1.5-2.2)
MH or SUD (11 457 560)	9.1 (7.9-10.3)	19.0 (17.8-20.2)	9.7 (8.9-10.5)	8.1 (7.4-8.9)	54.1 (51.6-56.5)

Abbreviations: ED, emergency department; MH or SUD, mental health or substance use disorder; NYU, New York University; PCT, primary care treatable; RFV, reason for visit.

Table 4 presents the most prevalent discharge diagnoses with corresponding reasons for visit, the NYU probabilistic assignment, and the probabilistic assignment based on the reason for visit at time of arrival at the ED. Overall, reasons for visit had higher probabilities of being classified across more categories using our method, particularly in injury-related, emergency care needed, and emergent but primary care–treatable categories, compared with similar discharge diagnoses’ classification using the NYU algorithm. For example, a discharge diagnosis of headache was classified as 77.9% nonemergent, 13.0% emergency care needed, and 9.1% emergent primary care treatable based on the NYU ED algorithm, whereas the reason for visit approach classified the visit as 45.6% nonemergent, 26.7% emergency care needed, 12.7% injury related, 11.8% emergent but primary care treatable, and 3.2% mental health or substance use disorder related. Among ED visits that resulted in hospital admission (inpatient), 52.6% were classified as emergency care needed, and 21.5% as emergent but primary care treatable using the NYU ED algorithm. However, the classification of the same visits based on the reason for visit indicated that 37.1% were encounters with needed emergency care and 26.6% were emergent but primary care treatable. Finally, among ED visits resulting in discharge diagnoses associated with stroke or heart attack that were classified as 100% emergency care needed and not avoidable using the NYU algorithm, less than half (47.0%) were similarly classified based on the presenting reason, with 39.3% of these encounters being classified as emergent but primary care treatable (21.7%) and nonemergent (17.6%).

**Table 4.** Probabilistic Assignment Comparison of the NYU and the RFV-Based Algorithms

Diagnosis	Probabilistic assignment category, %				
	Injury	ED care needed	Emergent but PCT	Nonemergent	MH or SUD
**Abdominal pain**					
NYU algorithm	NA	33.0	67.0	NA	NA
RFV algorithm	0.8	12.5	34.9	51.6	0.2
**Chest pain**					
NYU algorithm	NA	51.3	48.7	NA	NA
RFV algorithm	2.9	48.1	40.8	5.0	3.2
**Headache**					
NYU algorithm	NA	13.0	9.1	77.9	NA
RFV algorithm	12.7	26.7	11.8	45.6	3.2
**Low back pain**					
NYU algorithm	NA	11.1	15.3	73.6	NA
RFV algorithm	20.8	21.8	16.6	40.5	0.3
**Dizziness and giddiness**					
NYU algorithm	NA	8.0	20.0	72.0	NA
RFV algorithm	3.8	28.1	21.9	43.7	2.5
**Nausea or vomiting**					
NYU algorithm	NA	17.6	23.6	58.0	NA
RFV algorithm	2.5	27.9	29.6	37.3	2.7
**Cough**					
NYU algorithm	NA	11.8	23.5	64.7	NA
RFV algorithm	0.4	31.4	42.5	25.1	0.6
**Fever**					
NYU algorithm	NA	19.6	37.3	43.1	NA
RFV algorithm	0.6	24.3	38.9	36.1	0.1
**Disorders of teeth**					
NYU algorithm	NA	NA	NA	100	NA
RFV algorithm	3.8	12.3	34.7	49.2	NA
**Urinary tract infection**					
NYU algorithm	NA	24.1	29.7	46.2	NA
RFV algorithm	NA	27.2	30.7	40.6	1.5
**Hypertension**					
NYU algorithm	NA	21.0	17.7	61.3	NA
RFV algorithm	1.7	27.3	17.4	49.7	3.9
**Syncope and collapse**					
NYU algorithm	NA	66.7	33.3	NA	NA
RFV algorithm	7.3	53.9	26.7	6.6	5.5
**Dyspnea**					
NYU algorithm	NA	38.7	61.3	NA	NA
RFV algorithm	1.6	54.8	32.5	10.1	1.0
**Admitted to hospital**					
NYU algorithm	10.4	52.6	21.5	10.0	5.5
RFV algorithm	12.0	37.1	26.6	15.9	7.9
**Stroke or heart attack**					
NYU algorithm	NA	100	NA	NA	NA
RFV algorithm	7.6	47.0	21.7	17.6	6.1

Abbreviations: ED, emergency department; MH or SUD, mental health or substance use disorder; NA, not applicable; NYU, New York University; PCT, primary care treatable; RFV, reason for visit.

## Discussion

In this cross-sectional study using nationwide data of ED visits from 2018 and 2019 in the US, we developed an algorithm that assigned probabilities across 5 categories indicative of medical urgency based on presenting reasons for visit at triage. Our findings suggest that although the overall classification of ED visits based on post hoc discharge diagnoses and reasons for visit was similar (eg, 21.6% classified as nonemergent using the reason-for-visit approach and 21.8% using the NYU ED approach), a classification based on reasons for visit provided a wider distribution of probabilities across emergent and nonemergent categories, indicating that patients’ concerns and discharge diagnoses were not highly concordant. In addition, 53.9% of all ED visits were classified with high probabilities of falling within only 1 specific category using discharge diagnoses, while only 12.4% could be similarly classified based on the presenting reason for visit.

Our findings highlighted that that there was no association between presenting reasons for the visit at the time of arrival and the need for ED care and a final diagnosis.^23,24,25,28^ Medical complexity and the limited information available at the time of initial presentation to the ED based on patients’ concerns and/or symptoms make it challenging for clinicians to prospectively evaluate medical necessity and urgency.^23,24,25,28^ This was suggested and supported by our finding that even among discharge diagnoses defined and classified as very emergent, such as strokes or heart attacks, the initial reasons for visit for these conditions were similarly classified as emergent less than half the time (47.0%). This further illustrates the difficulty in making definitive assessments at the triage level based on the multiple possible outcomes associated with a presenting reason without first evaluating patients, with mistriage occurring in almost one-third of all ED encounters based on recent estimates.^32^ The uncertainty at the time of the encounter was also supported by our finding that only 3.1% of ED visits involving triage were deemed as nonurgent, which is in line with previous work.^24^

The diagnostic uncertainty at the time of arrival documented in our study suggests conceptual flaws of policies and cost-containment initiatives based on the premise that many ED visits are for medically nonurgent conditions and represent low-value care. With 0.4% of reasons for visits classified with full accuracy based on the presenting reason for visit, our findings question frameworks designed to redirect patients from the ED to other settings upon arrival. Our results are also supported by previous findings examining the association of introducing modest ED copayments with changes in ED visits, which documented decreases in both low-acuity and high-acuity ED visits, particularly among individuals with lower socioeconomic status.^21^

Given the complexities of defining a visit as emergent or not, studies evaluating interventions to reduce less-urgent ED visits have yielded mixed results, and the evidence to reduce such visits remains inconclusive.^11,13,16,33^ The most promising initiatives have considered and proposed interventions at both the patient and system levels, such as the colocation of primary care or urgent care facilities with the ED, patient education, telephone triage systems, telemedicine, deferral or referral appointments, and enhanced access to primary care.^11,13,33,34^ However, many of these initiatives rely on making a decision after the patient is triaged and evaluated by a clinician during an ED visit. In some cases, patients will be required to make a second visit to another facility after being seen in the ED, which is an additional burden, particularly for patients who are resource constrained.^9^ Our study underscores the importance of developing systems to include valid presenting status information, such as chief concern, symptoms, reasons for visit, and other information like intensive care unit admission or mode of arrival. Rather than just relying on discharge diagnoses when assessing the urgency level of the ED visit, incorporation of additional information could enable the development of objective tools to assess an ED visit’s complexity, particularly since triage systems and protocols are not standardized across US hospitals.^28,32,35^

### Limitations

Our study has limitations. First, we relied on the NYU ED algorithm as the basis to classify reasons for visits, which might not have accurately captured the acuity of presenting reasons. Second, we used the first listed reasons for visit and diagnoses, which might have similarly overestimated or underestimated the urgency of each encounter. Next, although the listing and classification of presenting reasons for visit available in the data were conducted by trained staff, they were conducted using verbatim text, which might have resulted in misclassification. However, this should not have systematically affected our study results. We also note that we used only reasons for visit at triage to categorize visits by medical urgency at presentation and did not consider the immediacy-of-evaluation variable that was available in the data, due to the rate of missing observations across this field. However, such information as well as vital signs would not have enabled us to change the assignment of different probabilities across various urgency categories, since we based our approach on the NYU ED algorithm.

## Conclusions

In this retrospective, cross-sectional study of 190.7 million ED visits among adults aged 18 years or older in the US in 2018 and 2019, results highlighted the uncertainties that both patients and ED clinicians face when patients arrive at an ED based on presenting reasons for visit and chief concerns. After mapping all possible discharge diagnoses to the same reasons for visit, the study found that only about 1 in 10 reasons for visit could be prospectively categorized with high accuracy while also having limited concordance with discharge diagnoses. Policies and interventions to contain less medically urgent ED visits relying on retrospective discharge diagnoses are questionable, and alternative methods are needed to identify nonemergent encounters more accurately.

## Notes

Supplement 1. **eTable.** Most Prevalent Discharge Diagnoses and Reasons for Visit

Supplement 2.
Data Sharing Statement
