# Supplementary material for: Concordance in Medical Urgency Classification of Discharge Diagnoses and Reasons for Visit
Source: JAMA Netw Open. 2024 Jan 10;7(1):e2350522. doi: 10.1001/jamanetworkopen.2023.50522 (PMC10782231; doi:10.1001/jamanetworkopen.2023.50522)
Supplement: Supplement 1. — eTable. Most Prevalent Discharge Diagnoses and Reasons for Visit [file jamanetwopen-e2350522-s001.pdf]

Supplemental Online Content

Giannouchos TV, Ukert B, Wright B. Concordance in medical urgency classification of discharge diagnoses and reasons for visit. *JAMA Netw Open*. 2023;7(1):e2350522. doi:10.1001/jamanetworkopen.2023.50522

eTable. Most Prevalent Discharge Diagnoses and Reasons for Visit

This supplemental material has been provided by the authors to give readers additional information about their work.

© 2024 Giannouchos TV et al. *JAMA Network Open*.

eTable. Most Prevalent Discharge Diagnoses and Reasons for Visit

Most prevalent discharge diagnoses	%	Most prevalent reasons for visit	%
Chest pain, unspecified/other	5.5	Abdominal pain, cramps, or spams	7.2
Abdominal pain, unspecified	3.3	Chest pain or soreness	6.5
Pain in unspecified joint	2.3	Shortness of breath	3.7
Urinary tract infection	1.8	Nausea/Vomiting	2.9
Headache	1.7	Headache/pain in head	2.8
Pain in limb, hand, foot, finger, or toes	1.5	Back pain, ache, soreness, or discomfort	2.8
Syncope and collapse	1.3	Cough	2.6
Low back pain	1.3	Symptoms/problems relating to psychological and mental disorders	1.9
Hypertension	1.3	Vertigo - dizziness	1.8
Dyspnea	1.2	Side/flank pain	1.6
Dizziness and giddiness	1.2	Leg pain, ache, soreness, or discomfort	1.5
Acute upper respiratory infection, unspecified	1.1	Low back pain, ache, soreness, or discomfort	1.4
Unspecified injury of face and head	1.1	Fever	1.3
Nausea/Vomiting	1.0	Knee pain, ache, soreness, or discomfort	1.3
Pneumonia, unspecified organism	1.0	Injury due to accident, not known occurrence	1.3

© 2024 Giannouchos TV et al. *JAMA Network Open*.
