# Supplementary material for: Concordance in Medical Urgency Classification of Discharge Diagnoses and Reasons for Visit
Source: JAMA Netw Open. 2024 Jan 10;7(1):e2350522. doi: 10.1001/jamanetworkopen.2023.50522 (PMC10782231; doi:10.1001/jamanetworkopen.2023.50522)
Supplement: Supplement 2. — Data Sharing Statement [file jamanetwopen-e2350522-s002.pdf]

Data Sharing Statement

Giannouchos. Concordance in Medical Urgency Classification of Discharge Diagnoses and Reasons for Visit. *JAMA Netw Open*. Published January 10, 2024.
doi:10.1001/jamanetworkopen.2023.50522

Data
Data available: No

Additional Information
Explanation for why data not available: The data used in this study are publicly available
